# Development of antifungal fibrous ocular insert using freeze-drying technique

**DOI:** 10.1007/s13346-024-01527-8

**Published:** 2024-02-16

**Authors:** Hoda E. Teba, Islam A. Khalil, Rana M. Gebreel, Lamiaa I. Fahmy, Heba M. El Sorogy

**Affiliations:** 1 Department of Pharmaceutics, Faculty of Pharmacy and Drug Manufacturing, Misr University for Science and Technology, 12566, 6th of October Giza, Egypt; 2 Department of Microbiology and Immunology, Faculty of Pharmacy, October University for Modern Sciences and Arts, 12451, 6th of October Giza, Egypt

**Keywords:** Chitosan, Insert, Freeze-drying, Fluconazole, Candida, Ocular delivery

## Abstract

Candida species is one of the pathogenic fungi of the eye responsible for keratitis that frequently causes vision impairment and blindness. Effective treatment requires long-term use of antifungal drugs, which is opposed by the defensive mechanisms of the eye and inadequate corneal penetration. The objective of this study was to develop a carrier for prolonged ocular application of fluconazole (FLZ) to treat keratitis. FLZ was encapsulated into chitosan fibrous matrices (F1–F4) using different chitosan concentrations (0.02, 0.1, 0.5, and 1%w/v, respectively) by freeze-drying as a single-step technique. Studying the morphology and surface properties of the inserts revealed a porous matrix with fibrous features with a large surface area. Thermal stability and chemical compatibility were confirmed by DSC/TGA/DTA and FT-IR, respectively. Loading capacity (LC) and entrapment efficiency (EE) were determined. According to the in vitro release study, F4 (0.11 mg mg^−1^ LC and 87.53% EE) was selected as the optimum insert because it had the most sustained release, with 15.85% burst release followed by 75.62% release within 12 h. Ex vivo corneal permeation study revealed a 1.2-fold increase in FLZ permeation from F4 compared to FLZ aqueous solution. Also, in the in vivo pharmacokinetic study in rabbits, F4 increased the AUC_0-8_ of FLZ by 9.3-fold and its concentration in aqueous humor was maintained above the MIC through the experimentation time. Studies on cytotoxicity (MTT assay) provide evidence for the safety and biocompatibility of F4. Therefore, the freeze-dried FLZ-loaded chitosan fibrous insert could be a promising candidate for treating ocular keratitis.

**Graphical Abstract:**

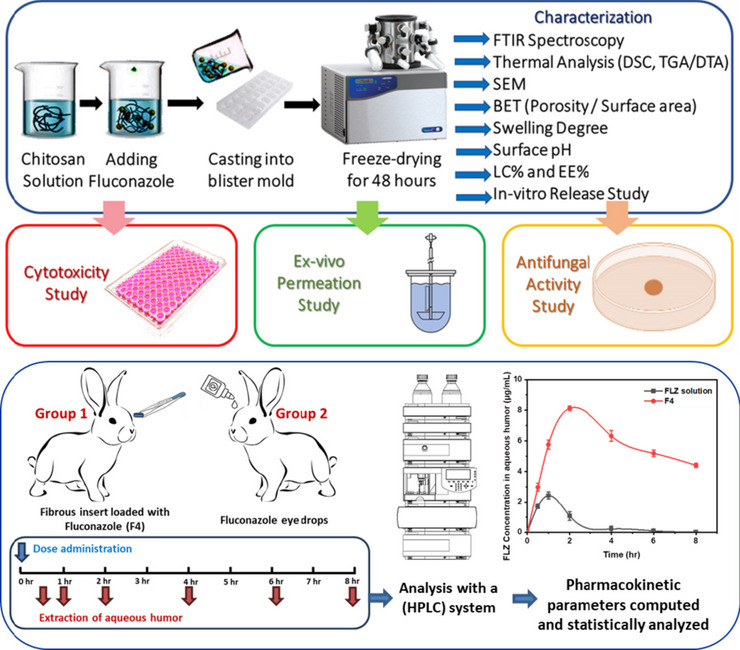

**Supplementary Information:**

The online version contains supplementary material available at 10.1007/s13346-024-01527-8.

## Introduction

Experts in drug delivery always have significant challenges in developing optimum drug delivery systems for the successful treatment of eye problems [1, 2]. The distinctive anatomy and physiology of the eye cause rapid clearance of the formulations from the surface of the cornea, which causes limitation of their corneal permeation. The majority of ocular treatments are available as eye drops and suspensions. Aqueous drops are unable to spread evenly on the surface of the eye due to their high surface tension [3]. As a result, only a small amount of drug (less than 5%) is absorbed from these conventional dosage forms, which would result in decreased drug levels below therapeutic concentrations [4]. Consequently, concentrated solutions and frequent instillation are necessary to reach an appropriate level of therapeutic efficacy. Many attempts have recently been conducted to improve topical ocular delivery by developing innovative drug delivery systems, such as liposomes, nanoparticles, nanoemulsions, nanosuspensions, micelles, and nanofibers [1, 5].

Keratomycosis, often known as fungal keratitis, is a serious and difficult disorder affecting the eye that can cause irreversible vision impairment or even blindness [6]. Fungal infections are more common after organ transplantation, chemotherapy, and intensive care units. Also, eye injuries, surgery complications, and usage of topical steroids are among the causative factors. Additionally, people who use contact lenses without sufficient cleanliness, whether for medical or cosmetic reasons, have worsened the situation [7]. Candida species have been identified as the primary cause of eye fungal infections, and its treatment demands the use of antifungal medication for a long period [8]. The difficulty in treating fungal keratitis is referred to the defensive mechanisms of the eye and the inadequate corneal penetration of antifungal drugs. If the precorneal residence time of medications could be extended, ocular therapy for fungal infections would be markedly enhanced. Topical natamycin, flucytosine, amphotericin B, and miconazole are used to treat fungal keratitis.

Fluconazole (FLZ) is one of the most effective antimycotic drugs belonging to the triazole family, which is very slightly soluble in water. It has valuable antifungal activity against a wide range of fungus species, including *Candida albicans* [9]. It exerts its antifungal activity by selective inhibition of ergosterol steroid that presents only in the cell membrane of fungi. However, its poor aqueous solubility, in addition to the rapid drainage, hinders its use in the form of eye drops. Also, its corneal permeability is limited and shows low ocular bioavailability because it has a low log *P* value (0.56) and a short half**-**life (15–30 min) [10, 11].

FLZ was previously developed into several topical ocular formulations to overcome the limitations of eye drops as liposomes [12], cubosomal nanoparticles [13], niosomes [14], and nanoparticles [15]. By using nanoparticles, patients do not need to administer the formulation frequently as they show sustained drug release. However, most nanoparticles are just as eye drops and have short precorneal residence duration. Hence, the incorporation of the nanoparticles in mucoadhesive polymeric inserts may be required to overcome their rapid elimination of nanoparticles from the corneal surface [16]. In addition to providing a prolonged release that extends the drug’s ocular residence time and increases its bioavailability, which may be achieved by using mucoadhesive nanoparticles, the polymeric ocular inserts provide accurate dose and can regulate the administration of therapeutic concentrations of the medication within the target tissues [17].

Ocular insert is a solid dosage form mainly used to treat ocular diseases that require a good contact time and sustain release drug profile. Compared to conventional carriers, ocular inserts will come into contact with conjunctival tissue for a long time, enhance bioavailability, and prolong therapeutic activity. Besides, they have less chance of any sensitive reaction because they contain no preservatives; lower systemic absorption is also observed since the insert provides a precise dose that is entirely maintained at the administration site. The lower chance of ocular and systemic side effects, longer shelf life, and less administration frequency contribute to better patient compliance. Even though ocular inserts have several benefits, their main drawback is the feeling of a foreign object in the eye [18]. However, using the freeze-drying technique produces a soft and delicate fibrous insert that is highly comfortable to be placed into the lower eyelid of the eye [19].

Many types of ocular inserts mainly depend on matrix solubility (insoluble, soluble, and bioerodible). Several techniques were used to fabricate ocular inserts, like solvent casting, melt extrusion, and direct compression. However, the freeze-drying technique is a promising technique, where it can produce ocular insert with a porous matrix structure directly from polymers without the requirement for structure-directing chemicals or pretreatments. The freeze-drying process is commonly known as lyophilization and can be considered a green method that is both environmentally safe and free of harmful substances [20]. Furthermore, the freeze-drying procedure is simple and economical and does not necessitate a high temperature or further leaching phase. As a result, it has gotten a lot of attention in fibrous matrix synthesis, starting with a solution, emulsion, or dispersion [21]. It was previously reported that freeze-drying can create a network of micro or nanofibers using a low concentration of polymer solution and a slow freezing procedure [22]. The freeze-drying process is completed in three steps: freezing, primary drying, and secondary drying. In the freezing step, a liquid sample is immersed in a cold bath or placed in a freezer, then the solvent crystals grow, and the solute molecules are excluded from the frozen solvent until the sample is completely frozen. After that, the frozen sample is placed in a freeze-drier (lyophilizer) to sublimate the frozen solvent, which is the drying step. The frozen sample should be kept below the glass transition temperature or melting point during the freeze-drying process, and the frozen solvent should be extracted under a vacuum. The spaces left when the frozen solvents are removed by sublimation are the structure’s pores [23, 24]. When the pressure is reduced, below the triple point, the frozen solvent sublimes resulting in primary drying. Secondary drying is used to desorb the unfrozen solvent attached to the polymer, and a lower vacuum level is employed to remove the bound water than in primary drying. Accordingly, the nanofibers prepared with the freeze-drying technique showed negligible cytotoxicity and good compatibility in the in vitro experiments, which are essential for their use in different applications [25].

It was previously reported that the use of polycationic mucoadhesive polymeric carriers (e.g., Chitosan) improved the contact time of ophthalmic preparations due to their ability to interact with the negatively charged cornea and conjunctiva via electrostatic interactions [26]. Chitosan (CS) is the N-deacetylated chitin polysaccharide derivative. It is the polymer of choice for ocular formulations due to its high biocompatibility, low toxicity, and biodegradability. CS can be employed to prolong the release of both hydrophilic and lipophilic drugs [27]. Furthermore, CS has proved to be effective in inhibiting the growth of a wide range of yeasts and fungi. Chitosan nanofibrous inserts can be used in applications that require controlled delivery as they are not soluble in water but are soluble only in a dilute acidic medium (pH < 6.5) [2]. Chitosan-based ocular inserts were reported to be well-tolerated in vivo with no signs of hemorrhage, intravasal coagulation, or hyperemia using the Hen’s egg test-chorioallantoic membrane (HET-CAM) irritation test [17].

This study aimed to develop a fluconazole-loaded CS polymeric fibrous ocular insert by freeze-drying technique to control drug releases, enhance drug penetration, and improve its antifungal activity for the treatment of fungal ocular infection.

## Materials and methods

### Materials

Fluconazole was kindly supplied by Sedico Pharmaceutical Company (Giza, Egypt). Chitosan (low Mw, viscosity, 20 cps, degree of deacetylation 85%) was purchased from Aldrich Chemical Co. (Stingham, Germany). Acetic acid, potassium dihydrogen phosphate, disodium hydrogen phosphate, sodium bicarbonate, calcium chloride, and sodium chloride were purchased from El-Nasr Pharmaceutical Chemicals Co. (Cairo, Egypt).

### Preparation of ocular insert

The CS concentration and loading of FLZ were studied as the main variables that may affect the formation of Fs at the same temperature. Aqueous chitosan (CS) solutions in various concentrations were prepared by dissolving a certain weight of CS in 2% (v/v) acetic acid to make a stock solution of 1% CS (w/v). The prepared 1% CS solution was then diluted to obtain various concentrations (0.02, 0.1, and 0.5 w/v %). The stock and the diluted chitosan solutions were poured into round-shaped blisters to be frozen at − 20 °C in a freezer and then freeze-dried for 48 h in a freeze-dryer (lyophilizer, Labconco, USA) to get plain formula. Each blister pack contains 1ml of CS solutions in various concentrations to produce uniform units of CS fibrous matrix for characterizations. For obtaining FLZ-loaded CS fibrous ocular insert, FLZ (0.3% w/v) was simply blended by magnetic stirrer (Model MSH-20D, GmbH, Germany) with each of the preformulated CS solutions prior to freeze-drying to obtain different fibrous ocular insert (F1, F2, F3, and F4) with different CS concentrations (0.02, 0.1, 0.5, and 1%), respectively.

### Physicochemical characterizations

#### Morphological examination

Scanning electron microscopy (Nova Nano SEM, FEI, USA) was used to examine the morphology of the CS fibers. The sample was placed on double-sided carbon tape in a vacuum chamber. The surface of the samples was scanned at 15 kV without previous treatment [28]. The average diameter of freeze-dried fibers was measured using image visualization software (Image J 1.45s, NIH Image, USA).

#### Surface properties

The nitrogen gas sorption analyzer (Quantachrome TouchWin_™_, USA) was used to measure the surface area, pore volume, and pore size of the plain and FLZ-loaded CS insert. These parameters were then calculated using the BET (Brunauer-Emmett-Teller) method at 77.35K. Samples were degassed in a vacuum oven at room temperature for 24 h before the BET measurement. With the Nova Enhanced Data Reduction Software, the BET surface area was calculated using the relative pressure range P/P° of 0–1. The calculation of pore volume and pore size was based on the Barrett-Joyner-Halenda (BJH) method.

#### Swelling degree

The FLZ-loaded chitosan ocular inserts, prepared with different CS concentrations, with known weight (5 mg), were immersed in phosphate buffer saline (pH 7.4) using a shaking incubator at 37 °C and 50 rpm. After 24 h, the swollen inserts were removed and placed on filter papers to remove excess surface water before reweighing them. To investigate the swelling behavior of the inserts, the percentage of swelling degree was calculated from the following equation [29].

1 $$\mathrm{Swelling\;degree\;}\%= ({{\text{W}}}_{{\text{s}}}- {{\text{W}}}_{{\text{d}}})/{{\text{W}}}_{{\text{d}}} \times 100$$

where W_d_ is the weight of dry inserts, and W_s_ is the weight of the insert after swelling. Each test was carried out in triplicates.

#### Surface pH

To measure the surface pH of the fibrous ocular inserts, they were placed in a Petri dish containing distilled water and given enough time (30 min) to swell at room temperature. The pH paper was left on the surface of the insert for 1min, and the produced color was compared to a standard color scale [30].

#### Loading capacity and entrapment efficiency

Each ocular insert (each insert equivalent to 1 ml of the CS/FLZ solution with different concentrations of CS) was suspended in 10 ml of 2% (v/v) acetic acid aqueous solution and placed in a shaking incubator for 24 h [17, 31]. After the CS ocular insert had fully dissolved, the maximum amount of FLZ released in the supernatant was measured using a UV spectrophotometer (UV spectrophotometer; Shimadzu, USA) at *λ*_max_ of 260.8 nm. The loading capacity and the entrapment efficiency of FLZ in the freeze-dried CS ocular insert were calculated as follows:

2 $$\mathrm{Loading\;Capacity\;}\%=({\text{A}}-{\text{B}})/\mathrm{C }\times 100$$

3 $$\mathrm{Entrapment\;Efficiency\;EE\;}\%=({\text{A}}-{\text{B}})/{\text{A}}\times 100$$

where A was the total amount of FLZ added, B was the free amount of FLZ in the supernatant solution, and C was the mass of the ocular insert. Each experiment was repeated 3 times. The average values and standard deviations for each experiment were calculated with statistical analysis.

#### In vitro release study

##### Determination of percentage of release

A known weight of each ocular insert (5 mg) was immersed into 10 ml of PBS of pH 7.4 in a shaker incubator that was adjusted at 37 °C ± 0.5 °C and 50 rpm for 12 h [32]. At certain release time intervals, 2 ml buffer solution was taken out, and the concentration of FLZ was determined spectrophotometrically at *λ*_max_ of 260.8 nm (UV spectrophotometer; Shimadzu, USA). Fresh PBS was added back to maintain the original volume. All measurements were performed in triplicate. The cumulative amount of released FLZ was calculated using the following equation:

4 $${{\text{Q}}}_{{\text{t}}} = {{\text{C}}}_{n}{{\text{V}}}_{{\text{t}}}+\Sigma\:{{\text{Q}}}_{{\text{n}}-1} {{\text{V}}}_{{\text{s}}}$$

where C_n_ is the concentration of FLZ at the releasing time t and *n* is the number of samples being taken for the UV analysis; V_t_ is the volume of the medium of release (PBS); Vs is the sample volume taken out for the UV detection. The percentage of FLZ released was calculated as follows:

5 $${{\text{Q}}}_{{\text{t}}}/{{\text{Q}}}_{\infty }\times 100$$

where Q_∞_ is the amount of FLZ incorporated into the ocular insert. The % released of FLZ was plotted as a function of time to evaluate the behavior of release.

##### Kinetic analysis of release data

In order to predict the release mechanism of FLZ from different ocular inserts, additional analysis was performed by fitting the release data according to several kinetic models (zero order (Eq. 6), first-order (Eq. 7), Hixson-Crowell model (Eq. 8), Higuchi model (Eq. 9) and Korsmeyer–Peppas (Eq. 10) using linear regression analysis.

6 $${{\text{Q}}}_{{\text{t}}}={{\text{K}}}_{0 }\mathrm{ t}$$

7 $${\text{log}}{\mathrm{\;Q}}_{{\text{t}}}={\text{log}}{\mathrm{\;Q}}_{0}-{{\text{K}}}_{1\:}\mathrm{t }/2.303$$

8 $${{\text{Q}}}_{0}^{1/3}-{{\text{Q}}}_{{\text{t}}}^{1/3} =\mathrm{log\:t }+\mathrm{ log\:}\kappa$$

9 $${{\text{Q}}}_{{\text{t}}}={{\text{K}}}_{\mathrm{H\:}}{{\text{t}}}_{ }^{1/2}$$

10 $${\text{log}}{\mathrm{\;Q}}_{{\text{t}}}/{\mathrm{ Q}}_{\infty }=n\mathrm{log\:t }+\mathrm{ log\:}\kappa$$

where Q_t_ represents the amount of drug released at time t, Q_0_ represents the amount of drug initially present in solution, K_0_ represents the zero-order release constant, K_1_ represents the first-order release constant, and K_H_ represents the Higuchi dissolution constant. κ (kappa), in the Hixson-Crowell equation, is a constant indicating the surface-volume relation. Q_t_/Q_∞_, in the equation of the Korsmeyer–Peppas model (fitted with the first 60% of drug release data), is the proportion of the drug released at time t, Q_∞_ is the total amount of the drug released, k is a kinetic constant, and *n* is the exponent that determines the drug release mechanism [33].

The release mechanism is considered a Fickian diffusion, based mainly on drug diffusion, when *n* < 0.45, but if 0.45 < *n* < 0.89, the release mechanism is non-Fick diffusion. If the value of *n* > 0.89, it means that both diffusion and erosion were responsible for the release mechanism [34]. The model with the highest correlation coefficients (*r*^2^) between the observed and fitted data was chosen as the one with the best fit. The DDSolver software (DDSolver: An Add-In Program for Modeling and Comparison of Drug Dissolution Profiles) was employed for this analysis.

### Compatibility study

#### Fourier transform infrared spectroscopy

FTIR spectra of pure fluconazole, pure chitosan, plain ocular insert (1% CS), and FLZ-loaded CS fibrous ocular insert were recorded using the KBr disc method on an FTIR spectrometer (Shimadzu, Tokyo, Japan) to investigate the chemical compatibility of the drug (FLZ) and the polymer (CS) in freeze-dried ocular insert. Samples (2 mg) were prepared as KBr pellets and scanned against a blank KBr pellet background with a resolution of 4 cm^−1^, and wave-number range 4000–400 cm^−1^ at room temperature.

#### Thermal analysis (DSC/TGA/DTA)

DSC (Shimadzu DTG 60 H, Kyoto, Japan) was used to relate the physical performance of the drug-loaded sample after freeze-drying with its individual components (drug and polymer). The melting point of pure FLZ, pure CS, their physical mixture (1:1), and the FLZ-loaded CS insert were determined by accurately weighting each sample of 2–3 mg in an aluminum pan and heating them to 350 °C at a rate of 10 °C/min. The sample cell was purged with nitrogen at a rate of 40 ml/min throughout the measurement.

Thermogravimetric analysis (TGA) and differential thermal analysis (DTA) (Shimadzu DTG 60H, Kyoto, Japan) were used to assess the thermal stability of the optimum plain CS insert and FLZ-CS insert. The weighed samples were heated from room temperature to 600 °C at a constant rate of 10 °C/min in a nitrogen atmosphere to measure changes in sample weight as a function of temperature.

### Ex vivo permeation study

Transcorneal permeation of FLZ from the selected loaded-nanofibrous insert was studied, and compared to the prepared FLZ aqueous solution. Freshly excised bovine eyes that were obtained within 1 h of the animals being sacrificed were checked carefully for any corneal damage; damaged eyes were appropriately discarded. The healthy corneas were stored in PBS at 4 °C after washing with normal saline. In open-ended cylindrical tubes that had a cornea securely covering one end via gauze, each formulation of F4 and FLZ solution was placed to be in contact with the cornea that was positioned so that the innermost endothelium layer faced the permeation medium (diffusion area of 0.785 cm^2^), and the outermost epithelial layer faced the formulation within the tube. USP dissolution apparatus II (Schleuniger Pharmatron, Switzerland) was used to perform the experiment. The glass tubes were attached to the paddle shafts, and the cylinder position was adjusted to submerge the corneas with formulae in the permeation media (30 ml of STF, pH 7.4) that was maintained at 37 ± 0.5 °C and stirring rate of 50 rpm. The total time of the experiment was 6 h, and at certain time intervals, 2 ml aliquots were withdrawn and refilled with fresh STF each time. The amount of FLZ permeated per unit time was calculated from Eq. (11) as follows;

11 $${\text{Q}}={\text{C}_\text{n}}\text{V}+{\sum_{\text{i}=1}^{\text{i}=1n=1}}{\text{C}_\text{i}}{\text{V}_\text{i}}/\text{A}$$

where Q (μg/cm^2^) is the cumulative amount of the FLZ permeated, V is the volume of the diffusion medium, V_i_ is the volume of the sample, C_n_ and C_i_ are the drug concentrations in the diffusion medium, and the concentration of the withdrawn samples, respectively, and A is the area of diffusion (cm^2^). The absorbance was obtained by analysis of the withdrawn samples by a UV spectrophotometer at *λ*_max_ of 260.8 nm. This experiment was performed in triplicates.

The cumulative amount of FLZ per unit area (g/cm^2^) that has permeated through each formulation over time (h) was plotted to determine its permeability. The cumulative permeation graph’s slope was used to compute the steady-state flux (J_ss_) across the corneal tissue according to the following equation [35]:

12 $${\text{J}}_\text{ss}=\mathrm{Q}/\text{A}.\text{t}\lbrack\mu\mathrm{gcm}^{-2}\mathrm{h}^{-1}\rbrack$$

where Q (μg) is the amount of FLZ crossing cornea, A (cm^2^) is the diffusion area, and t (h) is the exposure time. The permeability coefficient, P, was calculated as the ratio of J_ss_ to the initial drug concentration in the formulation (C_o_), as expressed in Eq. (13).

13 $$\text{P}={\text{J}}_\text{ss}/{\text{C}}_0\lbrack\mathrm{cmh}^{-1}\rbrack$$

### Cytotoxicity study

The direct in vitro cell toxicity of the selected insert (F4) was assessed by determination of the cell viability percentage of both plain and medicated inserts using 3-(4, 5-dimethylthiazol-2-yl)-2, 5-diphenyltetrazolium bromide compound (MTT assay). According to ISO10993-5 recommendations, cell culture tests were carried out using the L929 mouse fibroblast cell line, a standard cell line for cytotoxicity testing [36]. The ocular inserts were sterilized by exposing each side to UV light for 15 min before testing. In a 96-well plate, L929 fibroblast cells were cultured (100 µl/well) at a density of 3 × 10^5^ cells/ml in DMEM (Dulbecco’s Modified Eagle Medium) and incubated for 24 h at 37 °C and 5% CO_2_ then the tested formula were added to the cells. The control (cells without ocular insert) was a row of 96-well plates. After the incubation period of 24 h, MTT solution (20 µl of 5 mg/ml stock solution) was added to the media in each well, and the cells were incubated for an additional 4 h. Subsequently, 200 µl of dimethyl sulfoxide (DMSO) was added to each well to dissolve the formed formazan crystals. The culture was gently agitated until a homogenous solution was achieved after 15 min of incubation. Using a multi-well plate reader (FLUOstar^®^ Omega, Germany), the absorbance of formazan solutions was measured at a maximum wavelength of 570 nm. All experiments were carried out in triplicate (mean ± SD, *n* = 3). The percentage of cell viability was determined from Eq. (14), and the grade of cytotoxicity was concluded from the percentage of cell viability as presented in Table 1 [37].

**Table 1:** Cytotoxicity grading according to the percentage of cell viability

**Grade**	**Cell viability **(**%)**	**Cytotoxicity**
0	≥ 100%	None
1	75–95%	Acceptable
2	50–74%	further evaluation by morphological analysis
3	25–49%	Unacceptable
4	1–24%	Unacceptable
5	0%	Unacceptable

14 $$\text{Cell viability }(\%)=\mathrm{absorption\; test}/\mathrm{absorption\;control}\times 100 \%$$

### Antifungal activity study

For the in vitro investigation of the antifungal activity of FLZ-loaded ocular insert against *Candida albicans*, a filter paper disc diffusion method was performed [38]. A sterile brush was used to apply a suspension of the Candida (0.5 MacFarland) onto a sterile plate of solidified Sabouraud dextrose agar (SDA). Each unit of the ocular inserts (FLZ-loaded F4 and plain F4) resulted from freeze-drying of 1 ml 1% CS solution, with and without FLZ, respectively. Three Petri dishes were used; in the 1st one, sterile discs were immersed into an aqueous solution of FLZ (0.3% w/v), and were placed over the solidified SDA that was inoculated with Candida under aseptic conditions. The FLZ-F4 and plain-F4 ocular inserts were placed instead of the filter paper discs in the 2nd and 3rd plates, respectively. After 24 h of incubation at 37 °C, the zone of inhibition around the discs on the plates was measured. Each measurement was done in triplicate and was recorded.

### In vivo pharmacokinetic study

The ocular bioavailability of the selected fibrous insert loaded with FLZ (F4) and the FLZ aqueous solution was compared. Male New Zealand white rabbits were divided into two groups, with each group consisting of six rabbits. The average weight of these rabbits was between 2 and 2.5 kg. Rabbits were kept in typical housing conditions with regular 12-h light/12-h dark cycles at 25 ± 2 °C. Avoiding contact with the corneal surface, each rabbit in both groups (I and II) received 0.3 mg FLZ in the lower cul-de-sac of the right eye. This was achieved by applying 100 μl of FLZ solution in PBS pH 7.4 or the fibrous insert (F4) containing an equivalent amount of the FLZ, respectively. However, left eyes were considered as control by applying plain formulation. Upper and lower eyelids were held together for 10 s to improve the drug’s contact with the cornea. Eyes were anesthetized with topical application of 4% xylocaine solution, and at 0.5, 1, 2, 4, 6, and 8 h after the dose, a 22-gauge needle was carefully used for extraction of aqueous humor from the eyes to avoid their irritation. For precipitation of protein, 100 μl of methanol was added to 100 μl of the aqueous humor sample, refrigerated for 1 h, and centrifuged at 5000 rpm for 15 min. Then, samples were stored in a freezer (− 20 °C) for analysis with a high-performance liquid chromatography (HPLC) and quantitative estimation of FLZ in aqueous humor. A blend of deionized water and methanol (60:40 v/v) had been filtered and degassed to be utilized as the mobile phase with a flow rate of 1 ml/min and injection volume of 20 μl. The effluent was UV detected at 210 nm. To achieve consistent and accurate quantification, three solutions with known FLZ concentrations were employed as external standards. Caffeine (100 ng/ml) was added to the mobile phase as an internal standard. A total of 100 μl of the mixture was transferred to a glass screw-capped tube, followed by the addition of 50 μl of aqueous humor.

To study the ocular bioavailability of FLZ, pharmacokinetic parameters, namely, maximum drug concentration in the aqueous humor (C_max_) and time required to reach it (T_max_), were determined. Also, the area under the drug concentration–time curve from 0 to 8 h (AUC_0-8_) and mean residence time (MRT) were computed using WinNonlin software and statistically analyzed.

### Draize test and histological examination

It was carried out using three New Zealand white rabbits to evaluate the safety of the prepared fibrous insert and if there were any possible irritating effects. The right eye was considered as a control, with no treatment, while the left eye of each rabbit received the formulation (F4) at the bottom cul-de-sac twice daily. The eyes were checked for signs of ocular irritancy, such as redness, swelling, and watering of the eyes at specified time intervals (1, 24, 48, and 72 h) after the application of the inserts [39]. At the end of the Draize test, the rabbits that received F4 were slaughtered to perform the histological examination. The cornea was excised at the limbal edge, and after an instant washing with PBS, they were fixed with an 8% (w/w) formalin solution, dehydrated with alcohol, and washed with xylene. Then, they were placed in melted paraffin and hardened into blocks. Tissues were cut into 5-mm-thick slices, placed on glass slides, and stained with hematoxylin and eosin for examination with the light microscope.

### Statistical analysis

All results were statistically analyzed using GraphPad Prism version 6.00 for Windows (GraphPad Software, San Diego, CA, USA). One-way ANOVA was used to calculate the variance between and within each treatment. Statistical significance is indicated by (*p < 0.05, **p < 0.01, ***p < 0.001, and ****p < 0.0001).

## Results and discussion

### Evaluation of freeze-dried ocular insert

#### Morphological examination

**Fig. 1:**
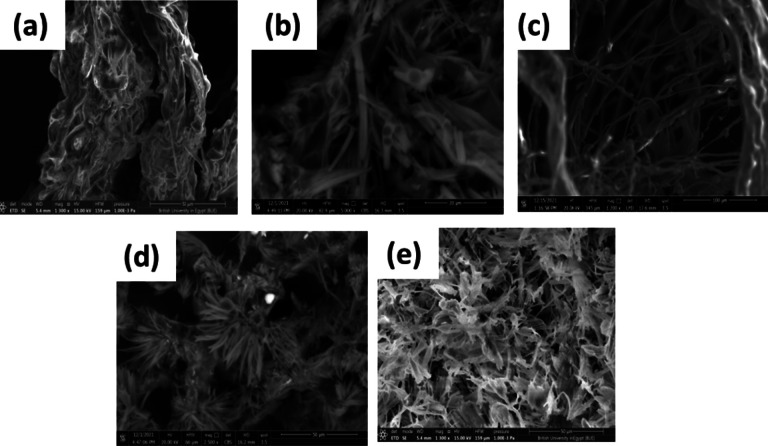
Morphological characterization of the fibrous ocular inserts using SEM techniques: (**a**) CS insert (0.02 wt% CS), (**b**) CS insert (0.1 wt% CS), (**c**) CS insert (0.5 wt% CS), (**d**) CS insert (1 wt% CS), and (**e**) F1 ocular insert (0.02 wt% CS and FLZ)

SEM was used to visualize the morphology of the freeze-dried ocular inserts. The effect of using different concentrations of CS (0.02, 0.1, 0.5, and 1 wt%) on the morphology of ocular insert and formation of a fibrous matrix by freeze-drying of CS solutions was studied (Fig. 1a–d). Generally, ice crystals formed when using a small amount of CS during the freeze-drying, and the polymer molecules of CS were excluded and then precipitated between them in a fibrous structure [40]. During the freezing step, the formation of fibers occurred in more than one stage; in the beginning, ice formed in the CS solution, but in this stage, the space between ice is not narrow enough to be at the fibrous level. Also, the polymer’s concentration between ices does not reach the solubility limit and does not precipitate. As more free water molecules transformed into ice, CS precipitated in the spaces between ices that reduced to a fibrous level. CS continued to concentrate and accumulate with the growth of ice over time. The production of fibers is complete when all water converts to ice. As previously reported, the morphology of the formed fibers is influenced by the polymer solubility and its concentration [41].

The morphology of the ocular insert with CS (0.02% w/v) showed a porous matrix with some fibrous features (Fig. 1a). At higher CS concentrations (0.1 and 0.5% w/v), the matrix showed a well-defined structure of nanofibers that were irregular in shape and interconnected together with an average diameter of 600–700 nm (Fig. 1b–c). However, 1% CS appeared as dendritic structures of nanofibers (Fig. 1d). It assumed that the large amount of CS molecules makes the distance between them small and might be aggregated and tend to clump together. This observation is consistent with that previously noticed by H. Yang et al., where the formed fibers were entangled in a block structure when a high concentration of PVA was freeze-dried [42]. Also, as previously reported, the molecular weight of the polymers used in the freeze-drying technique mainly affected the critical concentration at which the sub-micron fibers were produced [40]. Using CS with high molecular weight (Mw) resulted in nanofiber structure at concentrations of 0.02 and 0.1% rather than 0.5%. It may be referred to that to develop the nanofiber structure using a polymer with a higher Mw, a more diluted polymeric solution is necessary [2]. The same result was observed with PVA when the nanofibers formed at a concentration of 0.1% with high Mw; however, its formation with low Mw needed a 1% concentration. It could be explained by the fact that the polymer with a high Mw has longer chain molecules, which restricts the motion of the molecules [43, 44].

The effect of loading of FLZ at the CS concentration of 0.02 wt% was studied, where the addition of FLZ resulted in noteworthy changes in the morphology of the formed nanofibers with a significant decrease in their diameters (Fig. 1e). This result was consistent with that obtained by H. Iqbal et al. where the average diameter of the freeze-dried nanofibers decreased from 430 to 290 nm by loading of cefadroxil [45].

#### Surface properties

**Fig. 2:**
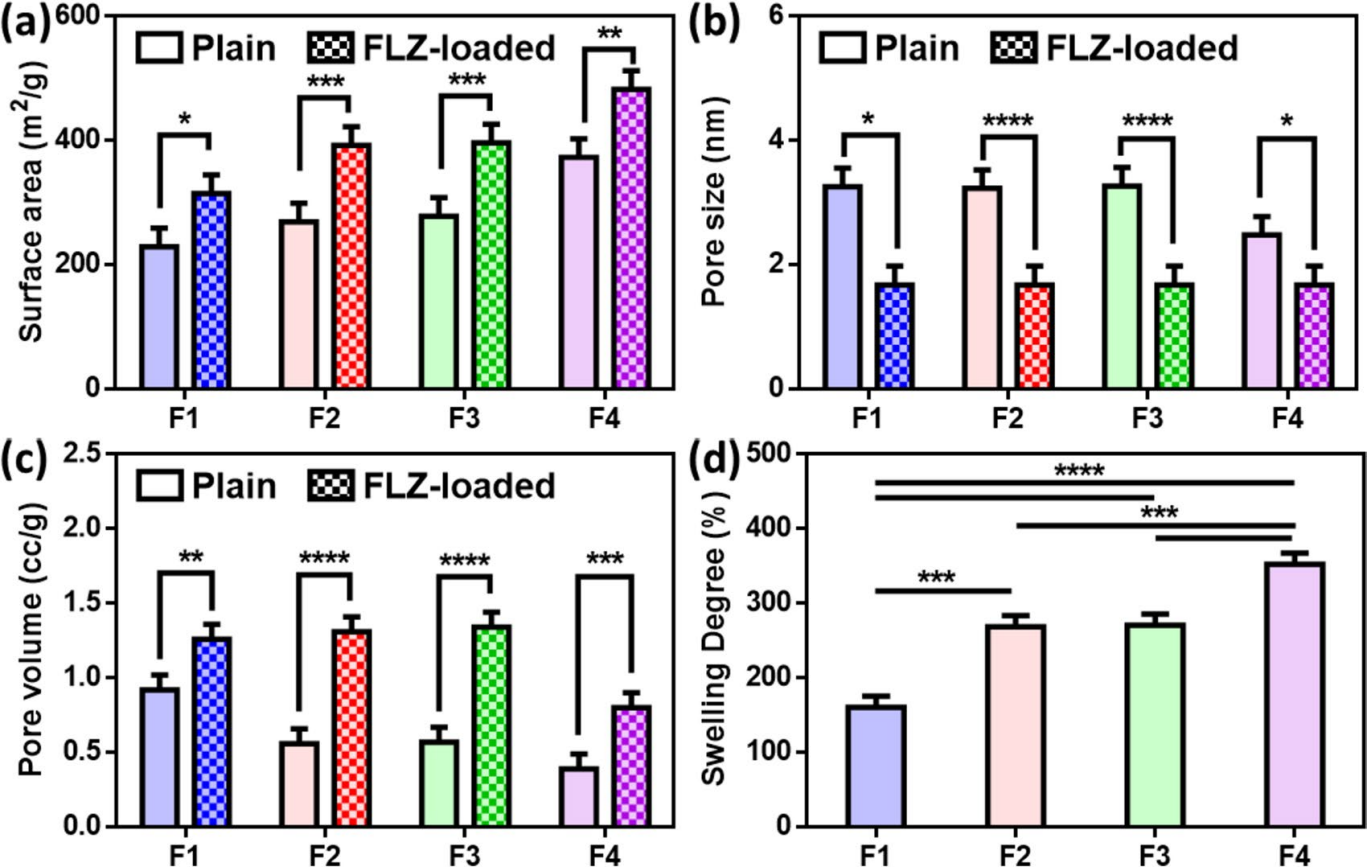
Brunauer–Emmett–Teller analysis of fluconazole-loaded freeze-dried ocular inserts (F1, F2, F3, and F4) with different concentrations of CS with different CS concentrations (0.02, 0.1, 0.5, and 1%), respectively. **a** Surface area, **b** pore size, **c** pore volume, and **d** swelling degree (mean ± SD, *n* = 3, *p < 0.05, **p < 0.01, ***p < 0.001, and ****p < 0.0001)

The nitrogen adsorption method was performed to determine the specific BET surface area, total pore volume, and pore size for the F inserts that were prepared with different concentrations of CS. Also, to study how these parameters were affected by the loading of FLZ. The results of the BET analysis are illustrated in Fig. 2a–c. Figure S1a–b shows the N_2_ adsorption–desorption isotherms and the pore size distribution curves obtained by the Barrett-Joiner-Halenda (BJH) method for different plain ocular insert formulations. Ocular inserts that were prepared with different concentrations of CS (0.02, 0.1, 0.5, and 1%) revealed a type IV-(a) isotherm that is specific to porous materials (according to the IUPAC classification) and indicated the presence of mesopores on the surface of plain inserts [46]. This mesoporous structure was confirmed by the average size of pores in all formulations. They were in the 2.48–3.27 nm range and were predictable due to the high BET surface area values [47]. A hysteresis loop is a characteristic property that reveals the presence of large numbers of pores in the mesoporous size [48]. All formulations exhibited H1 hysteresis loops that suggested the presence of cylindrical and conical mesoporous structures [48–51].

By comparing the isotherms of plain and FLZ-loaded ocular inserts, it was clear that the surface properties of the developed F inserts were affected by the loading of FLZ as the type of isotherms of the various formulations shifted from type IV to type V, as shown in Fig. S1c. It may suggest that the different formulations kept their porous structure even after loading of FLZ. In addition, the incorporation of FLZ caused a significant increase in the surface area of the freeze-dried F inserts (Fig. 2a), as the loading of FLZ resulted in fibers with a smaller diameter, which was confirmed by the images of SEM in Fig. 1e. For studying the effect of the polymer concentration on the surface properties, the BET results of the freeze-dried plain ocular inserts prepared with different concentrations of CS were compared (Fig. S1a) in addition to those of FLZ-loaded ocular inserts (Fig. S1c). It was noted that by increasing the CS concentration, the BET surface area of the formed fibers increased, but the pore size and pore volume decreased. Plain F1 (0.02 wt% CS) had the lowest surface area (229.34 m^2^/g) compared to 373.08 m^2^/g for F4 (1 wt% CS), which may result from its bulky structure, as shown in Fig. 2a. Furthermore, it was previously reported that the availability of water molecules from the surrounding solution had an impact on the formation of the ice crystals that mainly affect the pore size. When there was little CS present, water molecules could form ice nuclei and grow freely into large ice crystals. However, high CS concentrations resulted in a more dense gel network structure, which may have limited water molecule migration and ice crystal formation, resulting in smaller size of ice crystals and smaller pore sizes [52].

The pore size significantly decreased from 2.5–3.27 to 1.68 nm by the incorporation of FLZ (Fig. 2b). In the plain ocular inserts, the pore size decreased from 3.26 (F1) to 2.48 nm (F4) because the movement of solvent molecules during freezing might be affected by the viscosity of the polymer solution that increased by increasing the concentration of CS making it harder for the solvent molecules to concentrate and to arrange themselves which consequently developed smaller pores after the solvent crystals were eliminated, as previously reported by Z Cui et al. [53]. This result is consistent with Lei Qian and Haifei Zhang, as smaller pore sizes were observed when higher PVA concentrations were used [22].

The loading of FLZ increased the pore volume of all ocular inserts (Fig. 2c), which possibly would be attributed to the coverage of the surface area of the formed ice crystals by FLZ during the freezing step that could affect their growth capability leading to increment in the width of the spaces between ice crystals and resulted in larger pore volumes [54]. The decrease in the pore volume by increasing the CS concentration may be referred to that the pore structures between fibers are related to the ice crystal structures formed in the freezing step of the solvent. It is possible that F4 had less water and fewer ice crystals, and consequently, smaller pore volumes formed due to the removal of the ice during the freeze-drying process [2].

#### Swelling degree

Assessment of drug release from polymeric inserts and their bioadhesive capability is highly dependent on their swelling capacity. Upon insertion of the swelling-controlled system in the eye, aqueous solutions in the tear fluid diffuse inside the polymeric matrix, causing its swelling, which, in turn, induces polymer chain relaxation, drug dissolution, and diffusion. As the dispersed drug in the matrix will dissolve gradually, controlled release of the medication will occur. So, swelling of the polymer is the primary mechanism by which a soluble insert actually dissolves [55]. Furthermore, the polymers need to swell to start the formation of weak bonds that impart their bioadhesive properties [56]. After 24 h, the degree of swelling (%) of the four different fibrous insert formulations was calculated and compared, as illustrated in Fig. 2d. It is clear that the percentage of swelling degree increased as the concentration of CS increased, which was consistent with the previous studies, which stated that the higher the chitosan concentration, the higher the swelling degree [57, 58]. The high swelling capacity of CS in PBS refers to its hydrophilic nature due to hydroxy and amino groups, which give it a high affinity for salt solutions [18]. Also, it was claimed that the swelling degree of the CS ocular insert was directly related to the pore volume [2]. F1 (0.02% CS) showed the lowest degree of swelling (160.8 ± 6.32%), which may be attributed to the easy penetration of PBS into the ocular insert and its dissolution rather than its swelling, but still with good swelling behavior. As CS is poorly soluble and its amine groups remained deprotonated in PBS pH 7.4, and by increasing the amount of CS, more PBS might be absorbed in the outer layers of the system, and a stiff gel was formed that caused a slow dissolution of the system [59].

#### Surface pH

All of the CS Fs inserts showed surface pH values in between the range of 5 and 6, which established the suitability of the prepared inserts for ocular delivery without irritation [60–62].

#### Loading capacity and entrapment efficiency

**Fig. 3:**
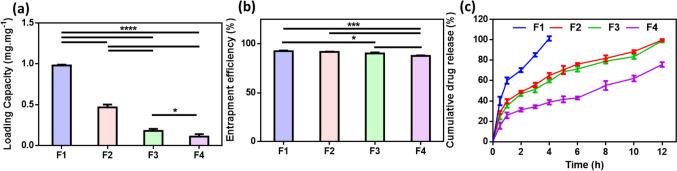
**a** Loading capacity (LC, mg mg^−1^), **b** entrapment efficiency (EE%) of FLZ-loaded ocular inserts, and **c** the drug release profile of the FLZ-loaded CS ocular inserts with different concentrations of polymer (CS) (mean ± SD, *n* = 3, *p < 0.05, **p < 0.01, ***p < 0.001, and ****p < 0.0001)

The drug loading capacity (LC) and entrapment efficiency (EE %) of FLZ from ocular inserts (F1–F4) prepared with different concentrations of CS were determined, and the results are presented in Fig. 3a–b. The difference in loading capacity was significant (*p* < 0.0001). LC decreased with increasing the CS concentration as the mass of the loaded ocular inserts was significantly increased. F1 showed the highest loading capacity (0.98 mg mg^−1^) compared to 0.47, 0.18, and 0.11 mg mg^−1^ for F2, F3, and F4, respectively. On the other hand, there was no significant difference in EE% between F1 vs F2 and F2 vs F3, but there was a significant difference between other formulations. The high percentage of entrapment efficiency of FLZ allows the usage of smaller inserts to attain its therapeutic effect, which is a valuable advantage of ocular inserts that will improve patient compliance.

#### In vitro release study

##### Determination of percentage of release

As previously reported, the drug molecules put into the ocular insert are released through different mechanism: desorption from the insert’s surface, diffusion through the pores, and fiber breakdown [63, 64]. Desorption of drug molecules that were adsorbed on the ocular insert’ surface was considered the principle cause of the initial burst release from the insert, in addition to their high specific surface area [65]. By the time, drug diffusion takes place as pores begin to form inside the ocular insert, as the release medium gradually penetrates them. Diffusion of drugs is the predominant mechanism and significantly contributes to its release along with other mechanisms, such as polymer swelling or polymer erosion in the biodegradable drug carrier system. The drug concentration gradient between formulations nanopores and their surrounding medium seems to cause the diffusion of drug molecules [66]. All the release profiles of FLZ from the different nanofibrous inserts (F1, F2, F3, and F4), as illustrated in Fig. 3c, exhibited a biphasic profile: the first phase of burst release followed by a sustained release phase. After 30 min of release, F1 was prepared with the lowest concentration of CS (0.02%) and showed 40.13 ± 4.2% burst release compared to 15.85 ± 3.13% for F4 (1% CS). F2 and F3 had 28.98 ± 1.41% and 24.74 ± 2.55% burst releases, respectively. F1 released about 100% of FLZ in 4 h, while in F2, F3, and F4, the % of FLZ released after 12 h was 99.71, 98.91, and 75.62%, respectively. It was clear that F4 had the most sustained release, which may be related to its highest concentration of chitosan [48]. Although it was expected for the F4 insert formulated with 1% CS to result in the highest FLZ release percentage as it was very hydrated and showed the highest swelling index (Fig. 2d), a reverse behavior was manifested, which may be referred to the porous nature of the inserts [34]. CS ocular insert may tend to associate together as their swelling progresses over time, and most of the pores are blocked as the fibers are merged by the hydrogen-bonding interaction between chitosan molecular chains due to excessive swelling, which results in a gel-like substance. Consequently, the drug diffusion path increased and hindered its diffusion. Therefore, the two membrane faces of the inserts are the only interface where drugs can diffuse [2, 67]. Hence, it could be deduced that the higher the swelling of the CS insert, the slower the release rate of the drug. Therefore, F4, which showed the most sustained drug release profile, was selected for further investigation, where this formula will positively influence patient compliance to treat fungal keratitis by minimizing the frequency of administration.

##### Kinetics of release data

In order to predict the release mechanism of FLZ from the ocular inserts, additional analysis was performed by fitting the release data according to several kinetic models (zero order, first order, Hixon-Crowel cube root, Higuchi diffusion, and Korsmeyer–Peppas model) using linear regression analysis. The correlation coefficients (*r*^2^) of each kinetic model are listed in Table 2.

**Table 2:** The correlation coefficients (*r*^2^) obtained from ocular inserts using different kinetic models and the “*n*” values obtained from Korsmeyer-Peppas model

**FLZ-loaded Fs code**			**r**^2^		**Korsmeyer-Peppas**	***n*** **value**
	**Zero order**	**First order**	**Hixson-Crowell**	**Higuchi diffusion**		
F1	0.811	0.850	0.891	0.910	0.942	0.380
F2	0.971	0.856	0.957	0.996	0.987	0.359
F3	0.970	0.879	0.955	0.993	0.988	0.415
F4	0.982	0.974	0.981	0.984	0.927	0.315

Zero-order equation is used for figuring out the medication release profile from different carriers when the drug release occurs at a constant rate. The first order explains the rate at which hydrophilic drugs are released from porous systems, with the rate proportional to the amount of the drug still present inside the carrier. The Higuchi diffusion model assumes that drug release is controlled and takes place from the delivery system via diffusion. The Hixson-Crowell cube root model is often used when the drug release rate is proportional to the surface area of the delivery system. Korsmeyer–Peppas model describes the drug release from polymeric systems and is used to learn more about the type of diffusion mechanism that could be described by the exponent (*n*). The release mechanism matches the Fickian diffusion if *n* ≤ 0.45, whereas the *n* value between 0.45 and 1 suggests irregular transport of the drug as its release follows a non-Fickian model [68].

The Higuchi release model is related to porous materials and yields the maximum *r*^2^ value for all the ocular inserts. The data from the in vitro release study were moreover fitted to the Korsmeyer-Peppas model to learn more about the FLZ release mechanism. As shown in Table 2, the values of “*n*” were less than 0.45, indicating the predominance of the Fickian diffusion mechanism, and diffusion is primarily responsible for controlling the release of FLZ from the freeze-dried CS F inserts. Typically, Fickian diffusion occurs from a cylindrical formulation or a swollen polymer matrix [69]**.** The same result was obtained by studying the release of ciprofloxacin from poly (vinyl alcohol)/dextran nanofibers [70].

### Compatibility study

Fourier transform infrared spectroscopy (FT-IR) and thermal analysis (DSC/TGA/DTA) were performed to investigate any chemical interaction that might have occurred between the polymer (CS) and the drug (FLZ) and to study the effect of freeze-drying on the formed insert.

#### FTIR analysis

**Fig. 4:**
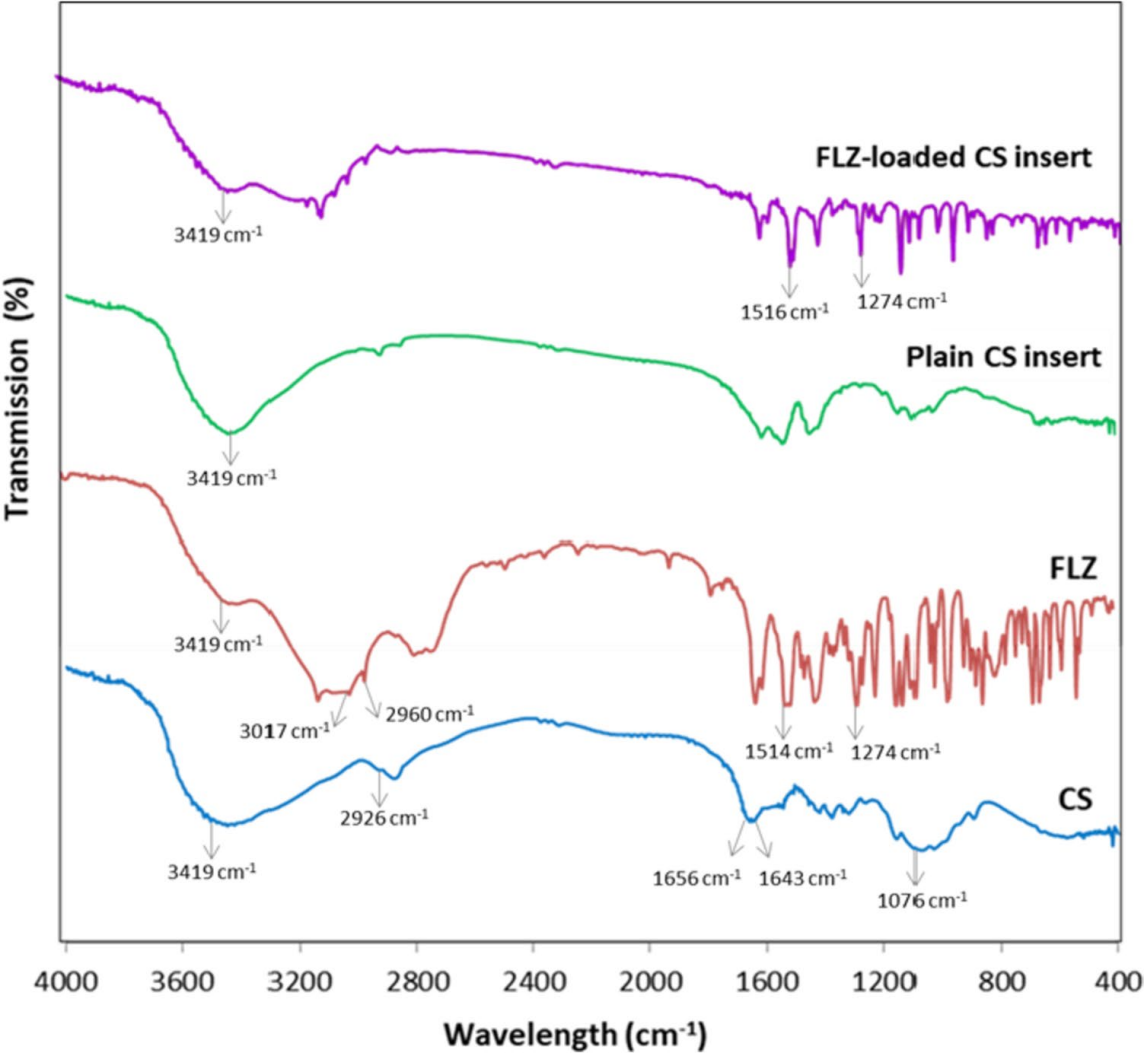
FT-IR spectra of chitosan (CS), fluconazole (FLZ), plain CS insert, and FLZ-loaded CS inserts

The FTIR spectra of the CS, FLZ, plain insert, and FLZ-loaded CS insert are displayed in Fig. 4. The spectrum of pure chitosan exhibited a broad band at 3419 cm^−1^ which corresponds to hydrogen bond N–H and O–H stretching vibration. Due to asymmetric and symmetric stretching, a band at 2926 cm^−1^ is seen, which is attributed to –CH_3_ and –CH_2_. The presence of C = O groups of acetylated amide and amine groups is confirmed by the development of peaks at 1656 cm^−1^ and 1643 cm^−1^. At 1076 cm^−1^, a band related to glycoside linkages was also observed [71]. The spectrum of FLZ showed a broad characteristic band of OH stretching at 3419 cm^−1^, characteristic bands of = CH and –CH at 3017 and 2960 cm^−1^, respectively, and the appearance of C = C aromatic stretching at 1514 cm^−1^ (C–F), stretching band appeared at 1139 cm^−1^, and a band at 1274 cm^−1^ due to (C-N) stretching of triazole ring. The breakdown of hydrogen bonds due to freeze-drying is clear in the spectrum of the plain insert and confirmed by the appearance of a sharp band of hydroxyl group when compared with the broad band spectrum of pure CS. The presence of FLZ characteristic peaks in the spectrum of the FLZ-loaded CS insert confirmed its existence inside the structure of the insert.

#### Thermal analysis (DSC/TGA/DTA)

**Fig. 5:**
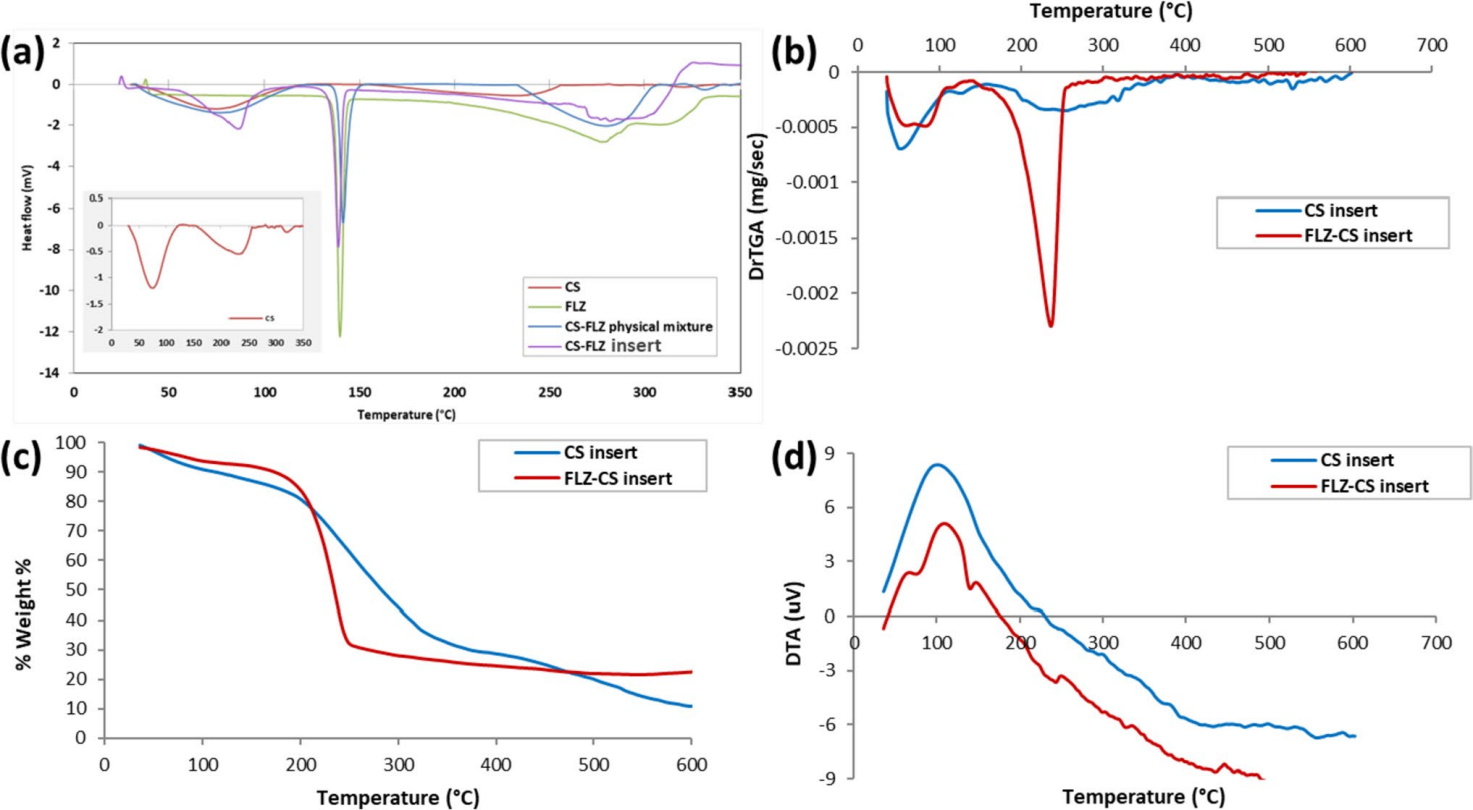
Thermal analysis: **a** DSC thermograms of pure chitosan (CS), pure fluconazole (FLZ), CS-FLZ physical mixture (1:1), and FLZ-CS freeze-dried insert. **b** TGA, **c** DTGA, and **d** DTA of plain CS-Fs and FLZ-loaded CS inserts

Figure 5a shows the DSC thermograms of FLZ, CS, FLZ/CS physical mixture, and freeze-dried FLZ-loaded CS inserts. The thermogram of pure chitosan exhibited a broad endothermic peak at 74.28 °C (starts at 37 °C and ends at 111 °C) due to the dehydration process which indicated the incomplete drying of chitosan and there were some bound water molecules associated with the hydrophilic groups of chitosan that had not been eliminated throughout the drying process [72, 73]. It did not show an endothermic melting peak due to the presence of CS in its amorphous state [59], and its degradation was above 250 °C due to its chemical degradation involving the anhydroglucosidic ring’s dehydration, the de-polymerization, and the breakdown of the de-acetylated and acetylated chitosan units. The pure FLZ showed a sharp endothermic peak at about 139.68 °C corresponding to its melting point as an indicator of its crystalline nature and also had a broad exothermic peak at 277.18 °C indicating its degradation as previously reported in the literature [74, 75]. In the thermogram of the physical mixture of CS and FLZ (1:1), the endothermic peak of FLZ was maintained at 141.4 °C revealing its physical compatibility with chitosan. However, its proportion in the physical mixture resulted in a peak with lower intensity than the pure FLZ. In the thermogram of the FLZ-loaded CS inserts, one peak was found at 86.27 °C as a characteristic peak of CS, and the additional peak was observed at 138.82 °C, confirming the presence of FLZ in the freeze-dried ocular insert. The slight decrease in the melting point of FLZ, in the freeze-dried insert, and the lower intensity of its characteristic peak may be attributed to the amorphous state of the FLZ and might suggest the decrease of its crystallinity during the formation of the ocular insert [76, 15, 77].

Thermogravimetric analysis (TGA) was performed, and Fig. 5b–d are illustrated to demonstrate the TGA, DTG, and DTA curves of the freeze-dried Fs, respectively, to investigate their thermal stability before and after drug loading. From the TGA curves (Fig. 5b), the overall weight loss from plain CS inserts and FLZ-loaded CS inserts was 88.19% and 75.99%, respectively. The degradation of the insert occurred in three stages; the first mass loss was 18.68% and 10.48% in plain and FLZ-loaded insert thermograms, respectively. This stage starts from 36 to about 200 °C and was attributed to the evaporation of adsorbed water because the hydrogen bonds between water and the adsorbent were broken. The thermal degradation of CS in the ocular insert was indicated by the large mass loss (50.62% and 65.59% in plain insert and FLZ-loaded CS insert, respectively), which started above 200 °C and observed in the second phase. This observation was in agreement with Cui et al. who stated that chitosan begins to degrade at around 250 °C [53]. The higher weight loss that occurred in FLZ-loaded Fs in this phase may be due to the degradation of both CS and FLZ. Plain CS Fs showed a third phase of mass loss (18.71%) above 400 °C.

The DTG curves (Fig. 5c), which are the first-order derivative of thermogravimetric profiles, clarify the temperatures of the peaks that correspond to the maximum weight loss rate that was at 51.04 °C and 236.41 °C for FLZ-loaded CS insert. However, the DTG curve of plain CS insert showed three peaks at 53.24 °C, 255.62 °C, and 526.9 °C that are associated with the loss of free water, loss of bound water, and breakdown of chitosan chain, respectively [78]. As described in the literatures, CS powder shows its degradation at 290 °C, and it was clear that the freeze-dried CS Fs degraded at a lower temperature and lower thermal stability that was most likely owing to the presence of a minor amount of acetic acid, which induced the thermal degradation [2, 79].

The DTA curves (Fig. 5d) of both plain and FLZ-loaded CS insert showed exothermic peaks at the same temperature but with lower heating rates in the loaded Fs. This observation is because the DTA curve of the fluconazole should have a melting endothermic peak similar to its DSC results [80].

### Ex vivo permeation study

In order to study the effect of using freeze-dried ocular inserts on the ocular permeability of FLZ, this ex vivo permeation study was performed, using bovine corneas, for the optimum FLZ-loaded ocular insert (F4) in comparison with FLZ aqueous solution.

**Fig. 6:**
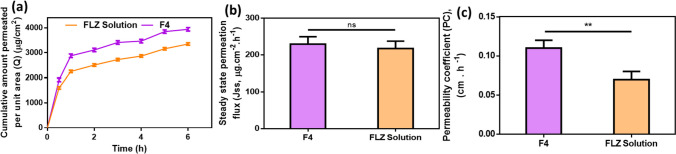
**a** Cumulative amount permeated per unit area, **b** permeation flux, J_ss_, and **c** permeability coefficient, PC, of FLZ from freeze-dried FLZ-loaded nanofiber insert (F4) and FLZ solution through the excised bovine cornea (mean ± SD, *n* = 3, **p < 0.01)

According to the results presented in Fig. 6a, where the cumulative amount of FLZ permeated was plotted against time, the ocular insert (F4) significantly (*p < 0.05) exhibited increased permeation than the aqueous solution containing the same concentration of FLZ (0.3% w/v) by 1.2-folds. The permeation parameters of FLZ from F4 and the aqueous solution were calculated, and the results are illustrated in Fig. 6b–c. The steady-state flux (J_ss_) was 229.27 ± 6.3 μg cm^−2^ h^−1^ and 217.42 ± 5.22 μg cm^−2^ h^−1^ for F4 and FLZ solution, respectively, indicating non-significant (*p* > 0.05) difference in their rate of permeation. However, the permeability coefficient (PC) was 0.11 ± 0.004 cm h^−1^ in F4, which is significantly (**p < 0.01) higher than FLZ aqueous solution (0.07 ± 0.002 cm h^−1^).

Large quantities of the drug on the surface, good wettability, and the presence of FLZ in nanosize in the fibrous matrix may all contribute to the higher permeation of FLZ from the ocular insert, in addition to the high surface-associated drug concentration in the fibrous matrix, aids in preserving sink conditions, and a gradient of concentration for drug diffusion [81]. Besides, as previously reported, due to the cationic structure, mucoadhesive nature, and penetration-enhancing properties of chitosan, the permeation across the mucosal epithelium was enhanced [82, 83]. The same result of improved permeation from the fibrous matrix was detected when Brinzolamide (BRZ) permeation through sheep corneas from BRZ-loaded nanofibers was compared with that of the commercial eye drops (Optilamid^®^) [84].

### Cytotoxicity study

In vitro cytotoxicity test by MTT assay was performed to study the effect of CS insert and FLZ-loaded CS insert on the viability of the cells. The assay depends on the release of dehydrogenase enzymes by the mitochondria of the living and active cells to convert the yellow tetrazolium salt to the water-insoluble purple crystals of formazan [85]. L929 mouse fibroblast cell cultures are the prevalent cell line employed in cytotoxicity assay due to their advantages in providing rapid quantifiable findings and relatively well-controlled factors [86]. Subsequently, they are regarded as a sensitive method of testing for cytotoxicity and were previously used in many studies when testing for ocular safety [87, 88].

**Fig. 7:**
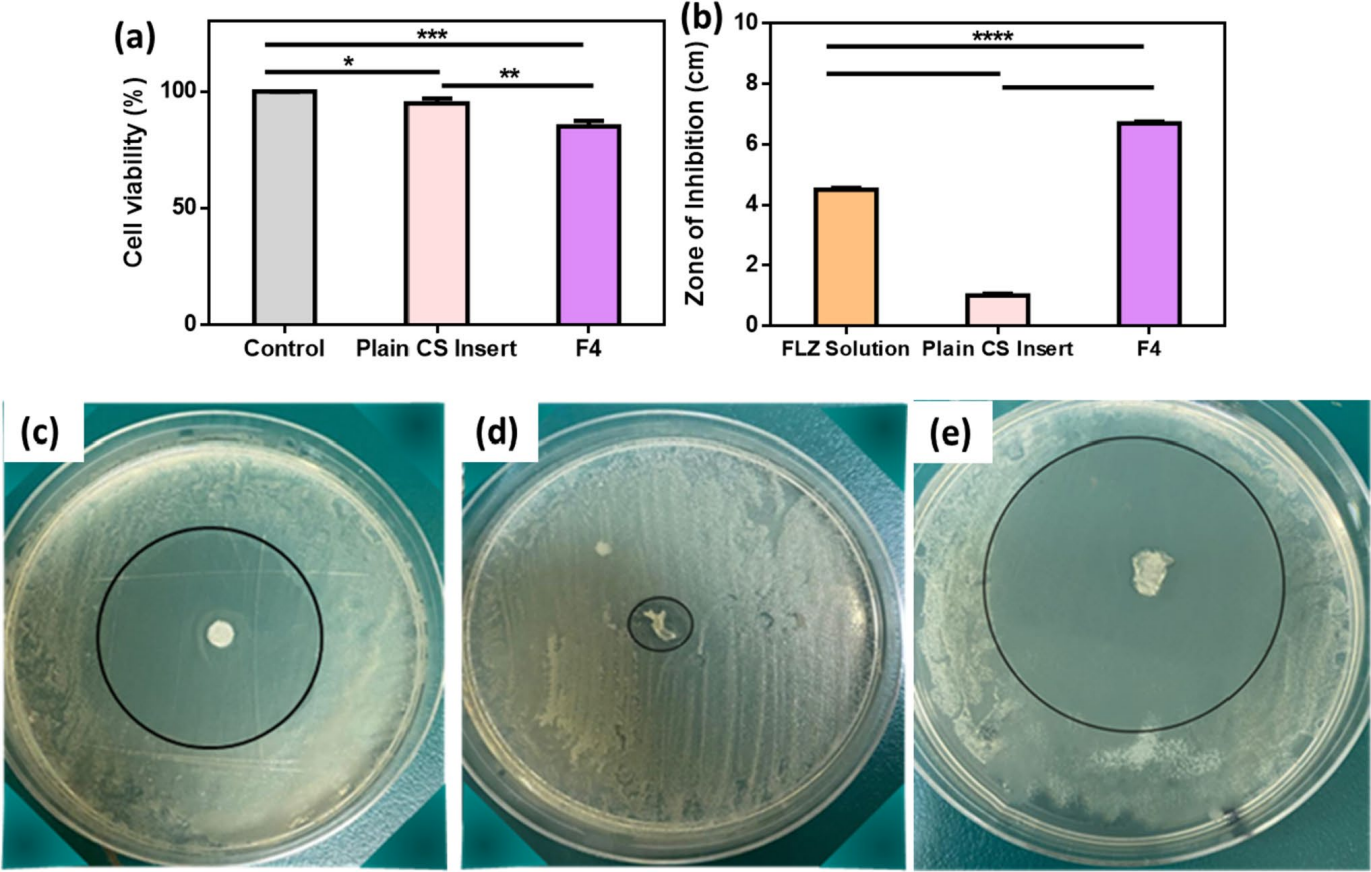
**a** Cell viability (%) obtained in MTT assays using L929 (fibroblast cells). **b** The antifungal inhibition zone of various formulations. The antifungal inhibition zone against *Candida albicans* of (**c**) FLZ aqueous solution, (**d**) plain CS ocular insert, and (**e**) FLZ-loaded CS ocular insert (mean ± SD, *n* = 3, *p < 0.05, **p < 0.01, ***p < 0.001, and ****p < 0.0001)

According to the absorbance values obtained from the MTT assay, the percentage of cell viability was calculated for each sample, and the results are illustrated in Fig. 7a. Both plain CS insert and F4 showed cell viability of more than 80%. Referring to the grading system for cytotoxicity, they belong to “grade 1” which is accepted for ocular application without irritation [37, 89].

### Anti-fungal activity

The antifungal activity of FLZ aqueous solution (0.3% w/v), FLZ-loaded ocular insert (F4), and plain CS nanofiber on the growth of *Candida albicans* was studied using the disc diffusion technique (Fig. 7b) which is the most used one among the various simple agar-based methods [90]. Results in Fig. 7c–e demonstrated that FLZ-loaded CS ocular insert (F4) showed a larger zone of inhibition (6.7 ± 0.05 cm) compared to an aqueous solution of FLZ (4.5 ± 0.06 cm), which means that it greatly inhibited the growth of *Candida albicans* and significantly enhanced the antifungal activity due to the synergistic effect between the FLZ and CS [91]. Similar finding was observed in previous studies of fluconazole-loaded CS nanoparticles for topical delivery [92] and of voriconazole-loaded chitosan nanoparticles [93]. The plain CS insert showed a small zone of inhibition, which may be attributed to the antifungal activity of chitosan. According to previous studies [94], it was reported that CS acts as a polycationic polymer at pH below 6.5 that proceeds its interaction with the negatively charged surface of fungi and causes their inhibition. Therefore, F4 is a promising ocular insert with controlled release and good permeation profiles, safe for corneal cells, and improved antifungal activity.

### In vivo study

#### In vivo pharmacokinetic parameters

**Fig. 8:**
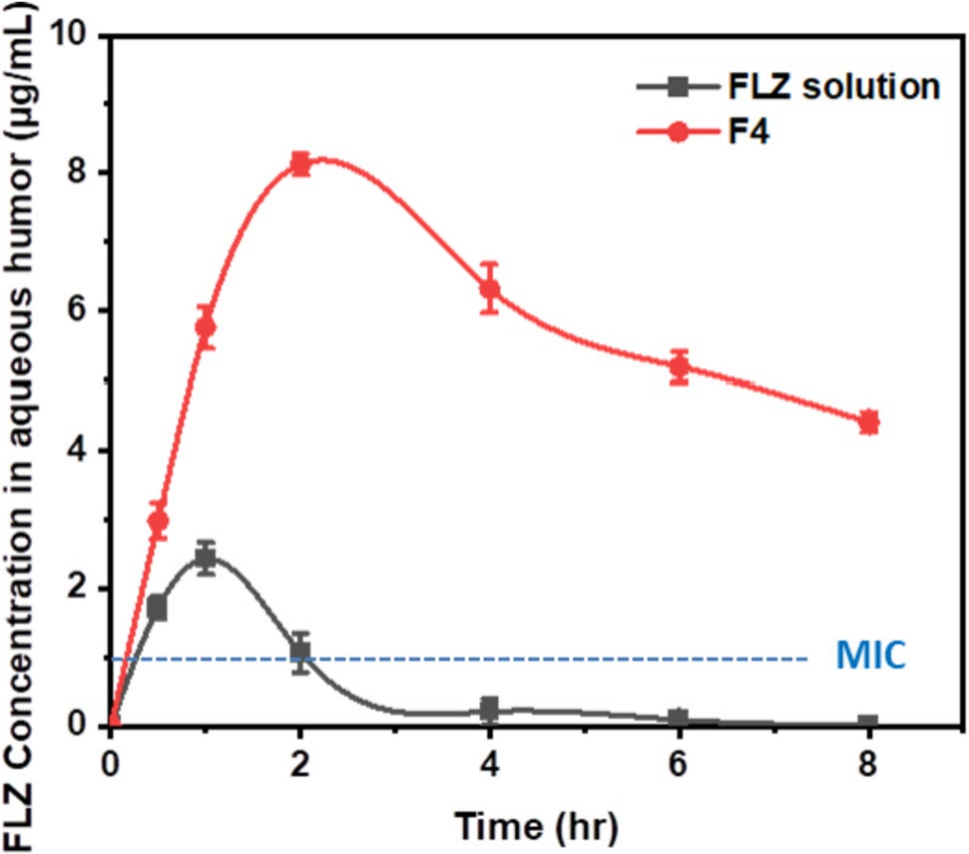
Concentration of FLZ in aqueous humor of rabbit’s eyes with time from the fibrous insert (F4) compared to FLZ eye drops (aqueous solution)

Table 3 represents the pharmacokinetic parameters of the selected fibrous insert formulation of FLZ (F4) and those of the aqueous solution of FLZ (eye drops). AUC_0-8_ is used as an indication for ocular bioavailability and showed a 9.3-fold increase for FLZ administered in fibrous insert (F4) compared to the aqueous solution. By applying one-way ANOVA, the difference among both formulations was statistically significant (***p < 0.001). In addition, the maximum concentration (C_max_) for FLZ in F4 was significantly higher than that of an aqueous solution by 3.3-fold. The minimum inhibitory concentration (MIC) range of fluconazole required for the successful treatment of candida species was previously reported to be 0.125–1 µg/ml [9]. It is clear that the concentration of FLZ in aqueous humor was maintained above the MIC through the experimentation time (8 h). On the other hand, as shown in Fig. 8, FLZ concentration was maintained above MIC for 2 h in the case of FLZ aqueous solution. This result was consistent with the calculated MRT, as F4 had a higher MRT by 2.5 times than the aqueous solution.

**Table 3:** Pharmacokinetic parameters of FLZ solution and FLZ-loaded fibrous insert (F4) in aqueous humor of rabbit’s eye

**Formulation**	**Pharmacokinetic parameters**				
	**C**_**max**_ **(μg/ml)**	**T**_**max**_** (h)**	**MRT** **(h)**	**AUC**_**0-8**_ **(μg h/ml)**	**Relative bioavailability (%)**
**FLZ solution (eye drops)**	2.43 ± 0.23	1	1.6 ± 0.16	4.87 ± 1.08	**-**
**F4 (fibrous insert)**	8.13 ± 0.16	2	3.96 ± 0.24	45.44 ± 1.11	9.33

The observed significant increase in FLZ pharmacokinetic parameters in the case of F4 could be attributed to the prolonged ocular residence time of the fibrous insert due to the nature of the dosage form and the excellent mucoadhesive properties of chitosan. In addition, the improved corneal permeability of FLZ from fibrous inserts could be another factor. As discussed previously in the ex vivo corneal permeability section, it was reported that the FLZ corneal permeability coefficient was increased 1.6 times from the fibrous insert with respect to the FLZ solution.

In conclusion, the abovementioned results suggest that FLZ fibrous insert has the potential to improve the therapeutic efficacy of FLZ for the treatment of fungal keratitis owing to prolonged ocular residence time offered by encapsulation of FLZ into the porous structure of fibrous matrix with a high swelling degree and excellent mucoadhesive properties.

#### Draize test and histological examination

**Fig. 9:**
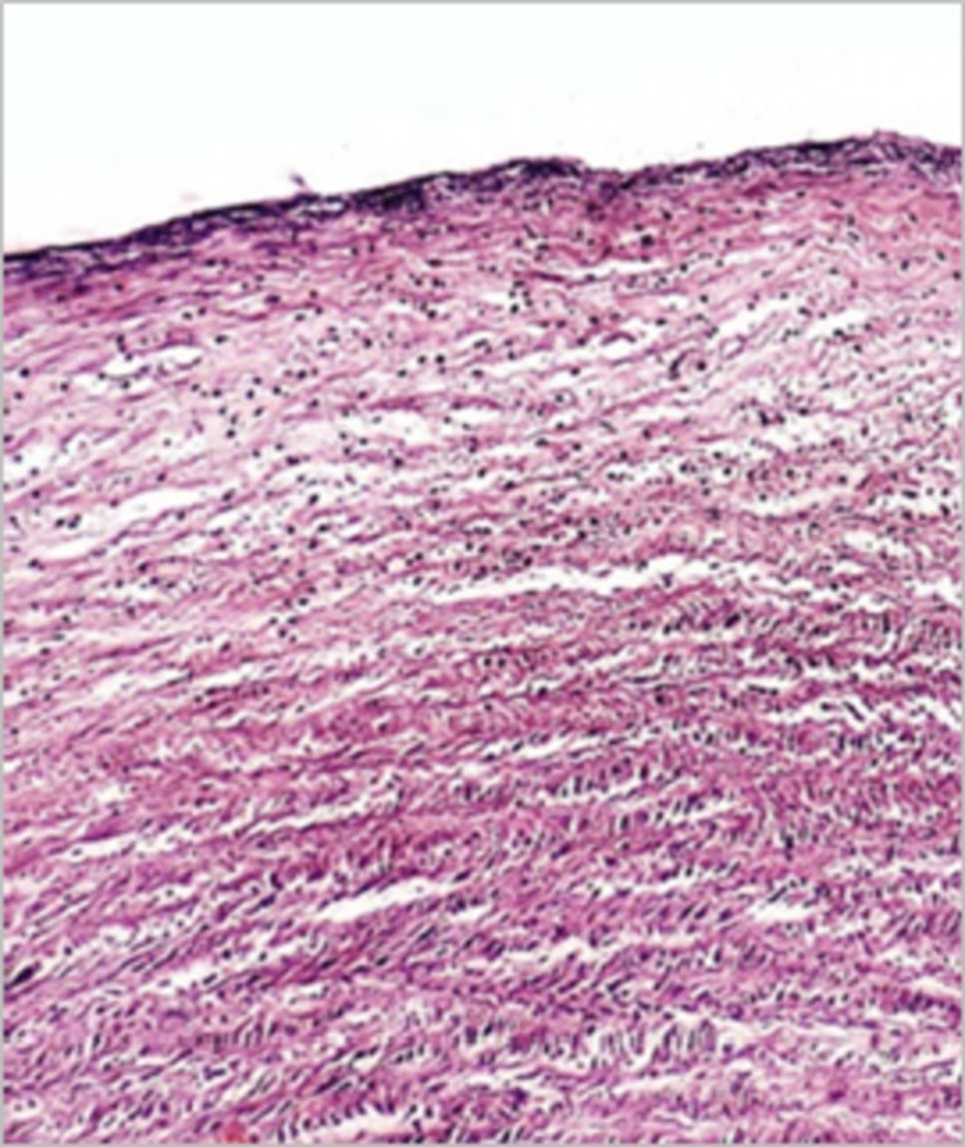
Histological examination: section of the excised corneal tissues treated with F4

Throughout this trial, no signs of ocular irritation, including redness, tearing, inflammation, or swelling, were seen in the rabbit’s eyes, proving that the formulation F4 was non-irritant and could be safely used in the eye. Further confirmation of the non-irritant effect of F4 was performed by removing the corneas from the eyes of the sacrificed rabbits that had previously received F4 in the Draize test. It showed normal histological features of the corneal layers with intact covering epithelium, underlying well-structured stromal collagen lamellar morphology, and intact posterior limiting membrane with lining endothelium, as shown in Fig. 9.

## Conclusion

This study confirmed the efficiency of the freeze-drying process as a method for the formulation of fibrous ocular inserts with a large surface area and small sheet-like platform that can positively influence patient compliance. Fluconazole was successfully incorporated as an antifungal drug and showed high entrapment efficiency. The current study proved that the formation of the fibrous matrix is highly dependent on the chitosan concentration. Furthermore, freeze-dried CS inserts can be considered a safe and efficient dosage form. The optimum ocular insert containing 1% chitosan (F4) was selected and compared with an aqueous FLZ solution. F4 showed more controlled release, better permeation across the excised bovine cornea, and a higher capability to inhibit *Candida albicans* growth. Also, F4 enhanced the pharmacokinetic behavior of FLZ in the aqueous humor compared to the FLZ solution without signs of irritation to the rabbit’s eyes. Therefore, F4 is a promising candidate for treating fungal keratitis.

### Supplementary Information

Below is the link to the electronic supplementary material.

Supplementary file1 (DOCX 319 KB)

## Data Availability

The datasets generated during and/or analyzed during the current study are available from the corresponding author on reasonable request.
