# Supplementary material for: Development of antifungal fibrous ocular insert using freeze-drying technique
Source: Drug Deliv Transl Res. 2024 Feb 16;14(9):2520–38. doi: 10.1007/s13346-024-01527-8 (PMC11291584; doi:10.1007/s13346-024-01527-8)
Supplement: Supplementary file 1 — Supplementary file1 (DOCX 319 KB) [file 13346_2024_1527_MOESM1_ESM.docx]

**Supplementary Data**

**Development of Antifungal Fibrous Ocular Insert Using Freeze-drying Technique**

Hoda E. Teba^a^, Islam A. Khalil^a^, Rana M. Gebreel^a^, Lamiaa I. Fahmy^b^ and Heba M. El Sorogy^a^

^a^ Department of Pharmaceutics, Faculty of Pharmacy and Drug Manufacturing, Misr University for Science and Technology, 12566, 6^th^ of October, Giza, Egypt

^b^ Department of Microbiology and Immunology, Faculty of Pharmacy, October University for Modern Sciences and Arts, 12451, 6^th^ of October, Giza, Egypt

Hoda E. Teba [hoda.teba@must.edu.eg](mailto:hoda.teba@must.edu.eg)

Islam A. Khalil [islam.khalil@must.edu.eg](mailto:islam.khalil@must.edu.eg)

Rana M. Gebreel [rana.mohamed@must.edu.eg](mailto:rana.mohamed@must.edu.eg)

Lamiaa I. Fahmy [Lismail@msa.edu.eg](mailto:Lismail@msa.edu.eg)

Heba M. El Sorogy [heba.moner@must.edu.eg](mailto:heba.moner@must.edu.eg)

**Corresponding author:** Heba M. El Sorogy

Egypt Email: [heba.moner@must.edu.eg](mailto:heba.moner@must.edu.eg)

Phone number: +201220892890, Egypt

Department of Pharmaceutics, Faculty of Pharmacy and Drug Manufacturing, Misr University for Science and Technology, 12566, 6^th^ of October, Giza, Egypt

| **F1** | **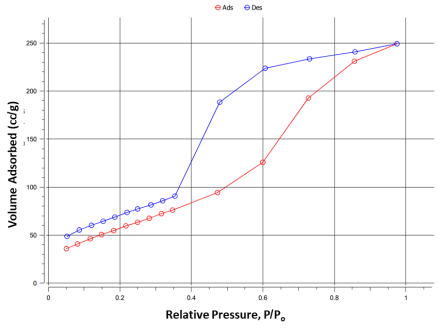** | **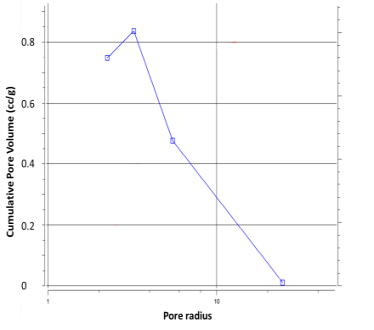** | 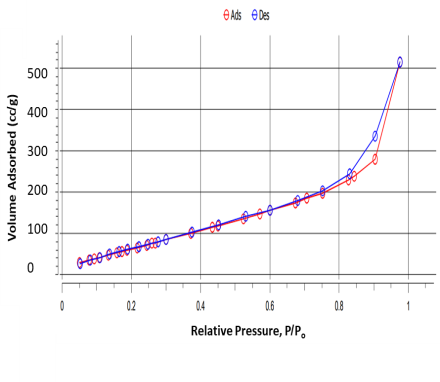 |
| --- | --- | --- | --- |
| **F2** | **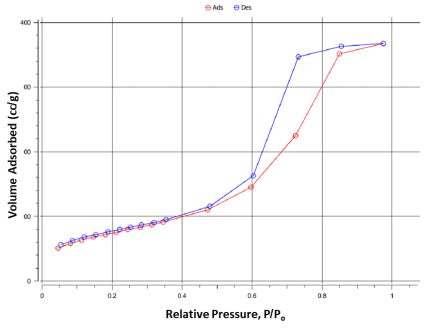** | **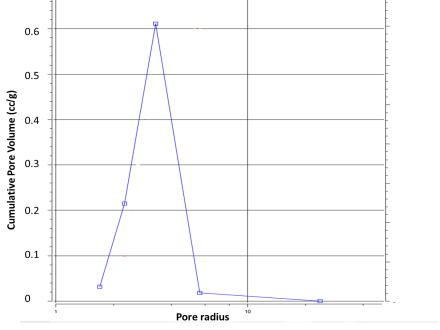** | **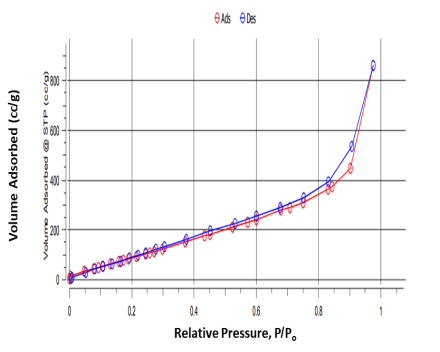** |
| **F3** | 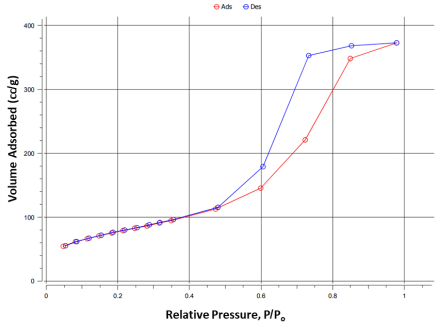 | **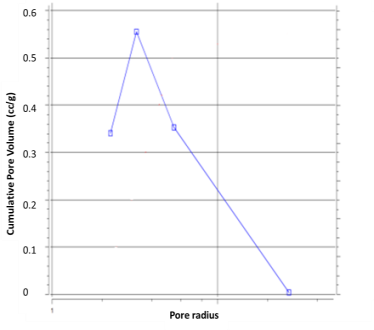** | **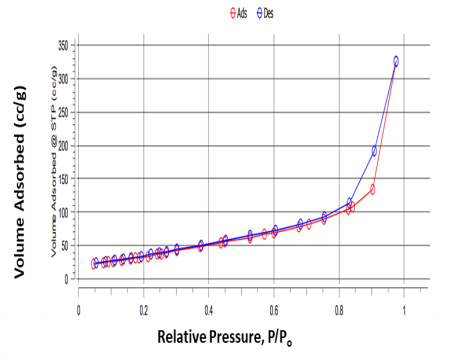** |
| **F4** | 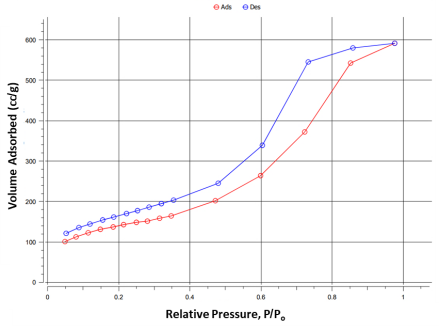 | 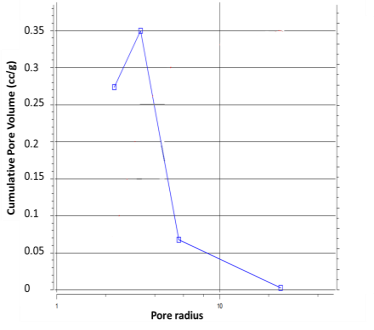 | **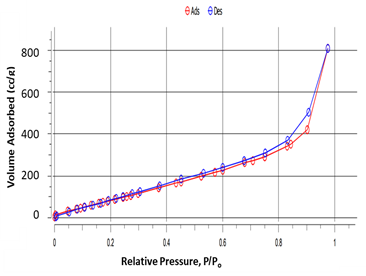** |
|  | **[a]** | **[b]** | **[c]** |

Fig. S1 N_2_ adsorption/desorption isotherms [a], The BJH pore size distributions of plain Fs [b], and N_2_ adsorption/desorption isotherms of FLZ-loaded Fs [c].
